# A 20-Year Longitudinal Study of *Plasmodium ovale* and *Plasmodium malariae* Prevalence and Morbidity in a West African Population

**DOI:** 10.1371/journal.pone.0087169

**Published:** 2014-02-10

**Authors:** Clémentine Roucher, Christophe Rogier, Cheikh Sokhna, Adama Tall, Jean-François Trape

**Affiliations:** 1 Laboratoire de Paludologie et Zoologie Médicale, Institut de Recherche pour le Développement, Dakar, Senegal; 2 Unité d'Epidémiologie, Institut Pasteur de Dakar, Dakar, Senegal; 3 Institut Pasteur de Madagascar, Antananarivo, Madagascar; Universidade Federal de Minas Gerais, Brazil

## Abstract

**Background:**

*Plasmodium ovale* and *Plasmodium malariae* have long been reported to be widely distributed in tropical Africa and in other major malaria-endemic areas of the world. However, little is known about the burden caused by these two malaria species.

**Methods and Findings:**

We did a longitudinal study of the inhabitants of Dielmo village, Senegal, between June, 1990, and December, 2010. We monitored the inhabitants for fever during this period and performed quarterly measurements of parasitemia. We analyzed parasitological and clinical data in a random-effect logistic regression model to investigate the relationship between the level of parasitemia and the risk of fever and to establish diagnostic criteria for *P. ovale* and *P. malariae* clinical attacks. The prevalence of *P. ovale* and *P. malariae* infections in asymptomatic individuals were high during the first years of the project but decreased after 2004 and almost disappeared in 2010 in relation to changes in malaria control policies. The average incidence densities of *P. ovale* and *P. malariae* clinical attacks were 0.053 and 0.093 attacks per person per year in children <15 years and 0.024 and 0.009 attacks per person per year in adults ≥15 years, respectively. These two malaria species represented together 5.9% of the malaria burden.

**Conclusions:**

*P. ovale* and *P. malariae* were a common cause of morbidity in Dielmo villagers until the recent dramatic decrease of malaria that followed the introduction of new malaria control policies. *P. ovale* and *P. malariae* may constitute an important cause of morbidity in many areas of tropical Africa.

## Introduction

*Plasmodium ovale* and *Plasmodium malariae* have long been reported to be widely distributed in tropical Africa and in other major malaria-endemic areas of the world [1]–[5]. In forest and wet savannahs areas of West and Central Africa, high prevalence of the two species is common in children, often reaching 15–40% for *P. malariae* and 4–10% for *P. ovale* in studies where thick blood films were carefully examined by trained microscopists since parasitemia is usually low [6]–[11]. In these areas with a long rainy season and/or perennial or semi-perennial *Anopheles* breeding sites, *P. falciparum* is always highly endemic, and most *P. ovale* and *P. malariae* infections are associated with *P. falciparum* infections [8], [12]. In the Sahel and other dry savannah areas, prevalence rates of *P. ovale* and *P. malariae* are much lower, rarely exceeding 1% for *P. ovale* and 10% for *P. malariae* [13].

The clinical features of *P. ovale* and *P. malariae* infections in endemic populations are poorly known, and most clinical data come from studies conducted in non-immune travelers returning from endemic areas or in patients that were given malaria therapy for the treatment of neurosyphilis during the period 1920–1960. High fever is the main symptom, attacks are mild and severe complications appear very rare [3], [4], [14], [15]. In malaria endemic areas of tropical Africa, almost all malaria attacks are attributed to *P. falciparum*, possibly due either to underdiagnosis of *P. ovale* and *P. malariae* clinical attacks, to partial cross-immunity between malaria species, or to rapidly acquired species-specific protective immunity against *P. ovale* and *P. malariae*. In the literature, there is little evidence that *P. malariae* may be responsible for fever episodes in children or adults living in highly malaria endemic areas, but the occurrence of fever episodes temporally related to peaks of *P. malariae* parasitemia was reported in Liberia [16], and the responsibility of this species in fever episodes occurring in children during the dry season has been suspected in The Gambia [17]. By contrast, the responsibility of *P. ovale* in fever episodes has been clearly established in Senegal both in children and in adults, but the incidence of the disease was much lower than for *P. falciparum* [18], [19].

In 1990, a longitudinal prospective study of malaria infection and the determinants of the disease was set up in the population of Dielmo village, a holoendemic area of Senegal [8]. Until 2008, when long-lasting insecticide-treated nets were deployed [20], the only intervention was to provide prompt specific treatment for malaria attacks and other diseases occurring in this village where the occurrence of fever cases was monitored daily. Here, we present parasitological and clinical data on *P. malariae* and *P. ovale* infections that we collected over a 20-year period in this population.

## Populations And Methods

### Ethics Statement

The project was initially approved by the Ministry of Health of Senegal and the assembled village population, and renewed on a yearly basis. Written informed consents were obtained individually from all participants or the parents of children younger than 15 years. For children participants aged 15–18 years, written informed consent were obtained individually since the National Ethics Committee of Senegal considered that participants in this age are responsible for their own person. Audits were regularly carried out by the National Ethics Committee of Senegal and ad-hoc committees of the Ministry of Health, the Pasteur Institute (Dakar, Senegal, and Paris, France), and the Institut de Recherche pour le Développement (formerly ORSTOM, Paris and Marseille).

### Clinical and parasitological monitoring

The study was carried out from June 1990 to December 2010 in Dielmo, a village situated in a Sudan-savannah region of central Senegal. It is an area of intense and perennial transmission where the mean entomological inoculation rate was 258 and 132 infected bites per person per year during 1990–2006 and 2007–2010 periods, respectively [20]. Most of the population of Dielmo (all 247 inhabitants of the village in June 1990 and 468 of 509 inhabitants in December 2010) was involved in a longitudinal study of malaria. The clinical, parasitological, entomological and epidemiological monitoring has been described elsewhere [8], [20]. Briefly, to identify all episodes of illness, a field research station with a dispensary was built and was open 24 h a day and 7 days a week for the detection of cases both active and passive. Each household was visited daily, and nominative information was collected at home 6 days a week (i.e. excluding Sunday) on the presence or absence in the village of each individual enrolled, their location when absent, and the presence of fever or other symptoms. The body temperature was recorded three times a week (every second day) in children younger than 5 years, and in older children and adults in case of suspected fever or fever-related symptoms (hot body, asthenia, cephalalgia, vomiting, diarrhea, abdominal pain, cough). In case of fever or other symptoms, thick blood films were made by finger prick and examined for malaria parasites, and medical examination and specific treatment were provided. In addition, thick blood films were made bi-weekly from June to September 1990, then weekly, monthly or quarterly according to periods and age-groups from October 1990 to December 2010 in all individuals enrolled in the project. Two hundred oil-immersion fields (approximately 0.5 µl of blood) were examined on each slide and the parasite: leukocyte ratio was measured separately for each plasmodial species. Since there was no simultaneous measurement of leukocytemia, when expressing the results in numbers of trophozoites per µl of blood, a mean standard leukocyte count of 8,000 per µl of blood was adopted for all age groups.

Four first-line antimalarial treatment were successively used during the 20 years study period: oral quinine (Quinimax®) (October 1990 – December 1994), chloroquine (January 1995 – October 2003), sulfadoxine/pyrimethamine + amodiaquine (SP+AQ) (November 2003 – May 2006) and artesunate + amodiaquine (AS+AQ) (June 2006 – December 2010). In most patients, antimalarials were given only in case of fever with a parasite/leukocyte ratio >2. At the beginning of the project, 48.6% (children: 51.1%, adults: 47.1%) of the villagers used traditional mosquito nets, which were untreated, and this proportion remained almost unchanged until July 2008 when long-lasting insecticide-treated nets (LLINs) were distributed to all villagers.

### Measurements of *P. falciparum, P. ovale* and *P. malariae* prevalence

Prevalence rates for each *Plasmodium* species presented in this study were those observed from 1990 to 2010 during bi-weekly (June–September 1990) or quarterly cross-sectional surveys (November 1990, then four quarterly studies each year from 1991 to 2010). All measurements of parasitemia (29,280) were taken into account, even if fever or other symptoms were documented at the same period.

### Establishment of criteria for the diagnosis of *P. ovale* and *P. malariae* malaria attacks

In persons living in areas where malaria is endemic, most *P. ovale* and *P. malariae* infections are asymptomatic. To distinguish the episodes of fever caused by *P. ovale* or *P. malariae* from those caused by other diseases when these parasites were present by chance, the relationship between parasitemia and fever was investigated by a case-control approach then the occurrence of age-dependent pyrogenic thresholds was investigated by a logistic regression method. A total of 64,284 simultaneous measurements of parasitemia and temperature made from June 1990 to December 2010 among 760 individuals aged from one month to 99 years were included in the analysis. The following definitions of case and control observations were used:

#### Case observations

Individual observations were regarded as fever cases if the rectal temperature measured by active case detection at home or by passive case detection at the clinic was ≥38°C (young children) or the axillary temperature was ≥37.5°C (older children and adults). Two fever episodes were considered independent if they occurred fifteen days apart or more. When several simultaneous measurements of parasitemia and temperature were available for the same fever episode, only the highest measure of parasitemia and the temperature associated were taken into account. 14,841 observations of parasitemia and temperature matched the case definition.

#### Control observations

Owing to the erratic nature of hyperthermia during malaria attacks and a number of other diseases, individual observations from the cross sectional surveys were considered to be asymptomatic controls if rectal/axillary temperatures were lower than 38°/37.5°C and if there was no episode of illness (allegation of fever and/or other fever related symptoms) between fifteen days prior and seven days after the temperature was taken. Measurements from pregnant women were excluded from the analysis. 49,443 simultaneous measurements of parasitemia and temperature performed during bi-weekly, weekly, monthly or quarterly cross-sectional surveys matched the control definition.

Since parasite prevalence and the occurrence of fever cases associated with the presence of malaria parasites decreased considerably during the most recent years, age-dependent pyrogenic threshold were investigated separately for the periods 1990–2004, 2005–2007, and 2008–2010. The method to calculate the age-dependent pyrogenic threshold by a random-effect logistic regression model of age and parasitemia was described earlier for *P. falciparum* parasitemia [21], [22]. Briefly the logit of the probability π_ij_ that the individual *i* presented a febrile episode during the observation *j* was expressed in the form of a linear function of age z_i_ and parasite density x_ij_. The best fit was obtained using a series of three dummy variables z_ik_ where k was an index coding for the four following age groups: 0–1, 2–6, 7–12, and ≥13 years old and using the *r*th power of x for the parasitemia. A threshold value in addition to the previous continuous effect of parasitemia was introduced as a binary variable s_ij_ and improved the goodness of fit of the model. The pattern of the threshold depending on age and parasitemia, the parameters used and the equation obtained was described previously [22]. β_0_ was a constant, α_i_ was the random-effects individual terms, β_1_, β_2_ and β_3_ were the regression coefficients.
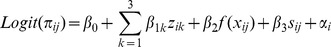



The models for each study period were compared according to the maximum likelihood (minimum deviance) using the Akaike method by minimizing the Akaike criterion [23]. All analyses were done with STATA software version 11.0 (College Station, TX, USA).

### Incidence density of *P. ovale* and *P. malariae* clinical attacks

For incidence density calculation, *P. ovale* and *P. malariae* clinical attacks were defined as any case with fever or allegation of fever and/or fever-related symptoms whose parasitemia was higher than the age and period corresponding threshold value of each species derived from the model (*P. malariae* all periods and *P. ovale* 1990–2004) or the case-control study (*P. ovale* 2005–2010). Two clinical attacks were counted separately if they occurred fifteen days apart or more. When *P. falciparum* associated parasitemia was higher than the age and period corresponding threshold of this species [22], the clinical attack to *P. falciparum* only was attributed, even in the few cases where *P. ovale* or *P. malariae* parasitemia thresholds were reached simultaneously. However, when distinct peaks of high parasitemia involving two malaria species were successively observed within fifteen days and the threshold was reached for each species, the fever episode was attributed to both malaria species.

## Results

### *P. ovale*, *P. malariae* and *P. falciparum* prevalences during cross-sectional survey

From 1990 to 2010, of 29,280 thick blood films realized during bi-weekly (June-September 1990) or quarterly cross-sectional surveys (from November 1990 to 2010), 14,341 (49.0%) were found positive for the presence of one or several malaria species, including 719 *P. ovale* (2.5%), 3,584 *P. malariae* (12.2%), and 13,216 *P. falciparum* (45.1%) infections with the parasite alone or in association. There were 2,912 mixed infections, including 2,320 *P. falciparum*/*P. malariae*, 314 *P. falciparum*/*P. ovale*, 12 *P. ovale*/*P. malariae*, and 266 *P. falciparum*/*P. malariae*/*P. ovale* associations.

**Figure 1:**
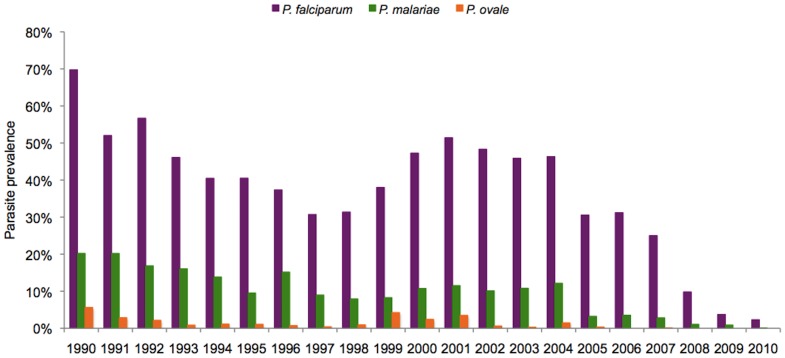
*P. falciparum, P. malariae* and *P. ovale* prevalence from 1990 to 2010 in Dielmo villagers (all age groups).

**Figure 2:**
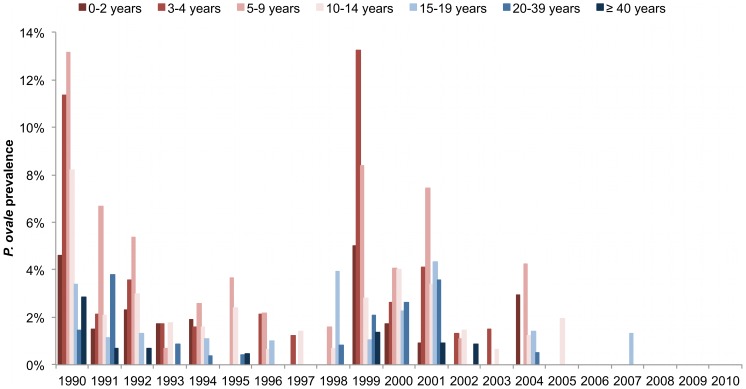
*P. ovale* prevalence by year and age group from 1990 to 2010.

**Figure 3:**
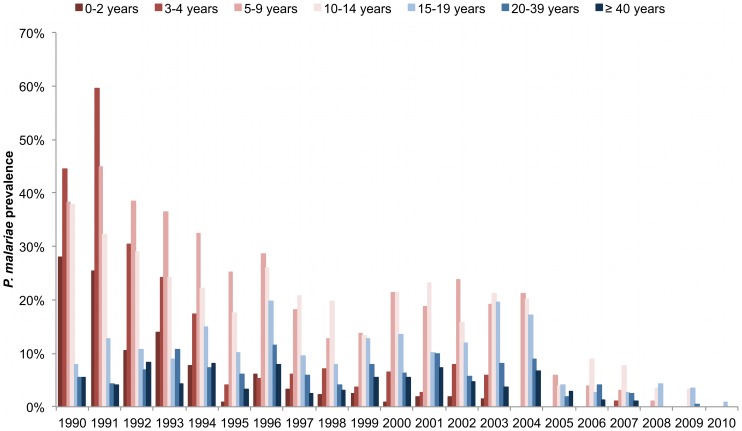
*P. malariae* prevalence by year and age group from 1990 to 2010.

Fig. 1 shows trends in malaria prevalence for each *Plasmodium* species from 1990 to 2010. *P. falciparum* prevalence was always higher than the prevalence of the two other species, and *P. malariae* prevalence was always higher than *P. ovale* prevalence. For all species, there was a marked decrease in prevalence during the most recent years. Fig. 2 shows *P. ovale* prevalence by year and age group. There were important annual variations in all age groups, with marked peaks in 1990, 1999 and 2001 reaching up to 7–13% in several age-groups in children. However, *P. ovale* almost disappeared after 2004. As shown in Fig. 3, *P. malariae* prevalence was very high (≈40%) in children during the first years of the project. The following years, prevalence declined markedly in young children, but remained high and almost unchanged until 2004 in older children and adults. There was a marked decrease in 2005–2009 and this species almost disappeared in 2010.

### Criteria for diagnosing *P. ovale* attacks

**Figure 4:**
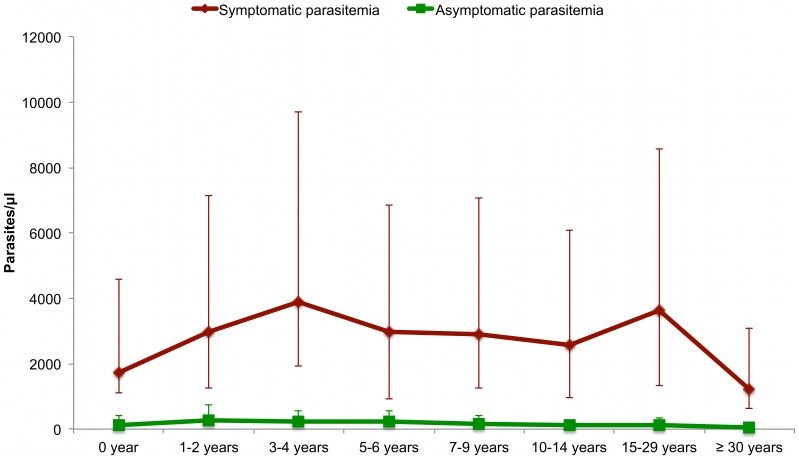
The mean *P. ovale* parasitemia (arithmetic mean of parasites per µl of blood) in asymptomatic control and fever case observations by age group, Dielmo 1990–2010.

Fig. 4 shows the mean *P. ovale* symptomatic and asymptomatic parasitemia according to age group between 1990 and 2010. Considering the changes in *P. ovale* prevalence during the 20 years of follow up, the relationship between *P. ovale* parasitaemia and the occurrence of fever was investigated separately during two different periods: from June 1990 to December 2004, and from January 2005 to December 2010.

The relationship between *P. ovale* parasitemia and the occurrence of fever during the period 1990–2004 is presented in Table 1. Of 12,182 episodes of fever, 598 (4.9%) occurred among patients infected by *P. ovale*. Of 37,074 observations among asymptomatic persons, 818 (2.2%) involved subjects infected by *P. ovale*. There was more fever cases with *P. ovale* associated with other *Plasmodium* species (412 cases, 68.9%) than fever cases with *P. ovale* only (186 cases, 31.1%). Risk of fever increased by 10-fold when *P. ovale* parasitemia was above 800/µl of blood (p<0.001). Above 4,000 parasites per µl of blood, only 3 (2.0%) *P. ovale* infections were asymptomatic and the 146 other infections (98.0%) presented fever. Maximum *P. ovale* parasitemia observed during a fever episode was 88,320/µl in a 4 year old child (second highest value: 36,000/µl in a 3 year child) and the maximum parasitemia without documented symptoms was 5,040/µl in a 4 year old child.

**Table 1:** Relationship between *Plasmodium ovale* parasitemia and risk of fever.

Level of *P. ovale* parasitemia^a^	Number of cases								
	Fever		Asymptomatic		Total				
	n	(%)	n	(%)	n	OR	95% CI	p^b^	
**1990–2004**									
0	11,584	(95.1)	36,256	(97.8)	47,840	1.0		-	
<80	265	(2.2)	627	(1.7)	892	1.7	[1.4–2.0]	**<0.001**	
80–399	43	(0.4)	95	(0.3)	138	1.2	[0.8–1.8]	0.285	
400–799	47	(0.4)	54	(0.1)	101	2.1	[1.4–3.3]	**<0.001**	
≥800	243	(2.0)	42	(0.1)	285	13.8	[9.7–19.6]	**<0.001**	
Total	12,182		37,074		49,256				
**2005–2010**									
0	2,655	(99.8)	12,359	(99.9)	15,014	1		-	
<800	1	(0.04)	8	(0.1)	9	0.7	[0.1–6.0]	0.749	
≥800	3	(0.1)	2	(0.02)	5	9.8	[1.4–68.4]	**0.021**	
Total	2,659		12,369		15,028				

a Parasites/µl of blood.

b Fisher's exact test. p values shown in bold are significantly associated with an increased risk of fever.

Dielmo, 1990–2004 and 2005–2010.

**Figure 5:**
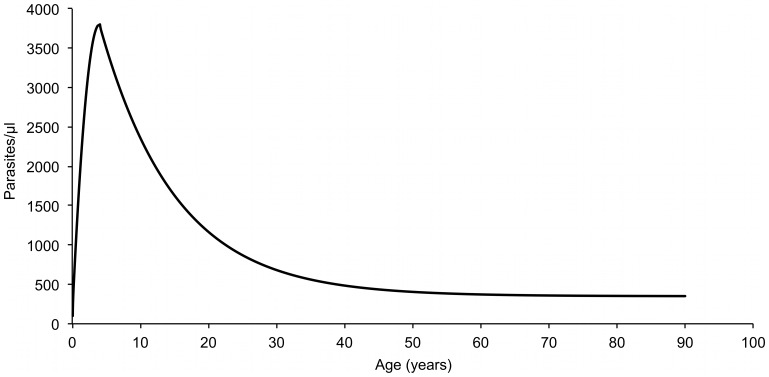
Random-effect logistic regression model derived threshold level of parasitemia for attributing fever episodes to *P. ovale* by age, Dielmo, 1990–2004.

Fig. 5 shows the age-dependent pyrogenic threshold levels of parasitemia during the period 1990–2004 are shown. Estimates of the parameters defining the age-dependent threshold are presented in Table 2. Highest threshold parasitemia in 4 year old children was 3,800/µl and lowest threshold parasitemia in adults was 350/µl.

**Table 2:** Estimates of the parameters defining the age-dependent pyrogenic threshold of *P. ovale* infections during the period 1990–2004.

Parameters	Period 1990–2004
Number of individuals	617
Number of case observations	12,182
Number of control observations	37,074
Exponent for the function of parasitemia (*r*)	0.52
Age in years of maximum parasitemia (*a*)	4
Maximum threshold parasitemia (*b*)	3800
Parasitemia at year 0 (*c*)	100
Lowest threshold parasitemia in adults (*d*)	350
Decay parameter of the function (*e*)	0.09
Threshold effect odds ratios (95% CI)	5.6 (2.2–13.9)
Continuous effect of parasitemia odds ratios for an increase of 100 parasites/µl (95% CI)	1.4 (1.2–1.5)
0–1 year old effect odds ratios	1.0
2–6 years old effect odds ratios (95% CI)	1.9 (1.8–2.1)
7–12 years old effect odds ratios (95% CI)	1.9 (1.7–2.1)
≥13 years old effect odds ratios (95% CI)	2.0 (1.7–2.3)
AIC	47315.0

The relationship between *P. ovale* parasitemia and the occurrence of fever during the period 2005–2010 is presented in Table 1. Of 2,659 episodes of fever, only 4 (0.15%) occurred among patients infected by *P. ovale* and three of these cases (adults 19, 30 and 33 year old, respectively, all with parasitemia >3,000/µl) corresponded to infections with *P. ovale* only, the fourth case (parasitemia 7/µl in a 7 year old child) being associated with *P. falciparum*. Risk of fever increased by 13-fold when *P. ovale* parasitemia was above 800/µl of blood (p<0.001). Of 12,369 observations in asymptomatic persons, only 10 (0.08%) involved persons infected by *P. ovale* and parasitemia ranged from 2 to 960/µl.

### Criteria for diagnosing *P. malariae* attacks

**Figure 6:**
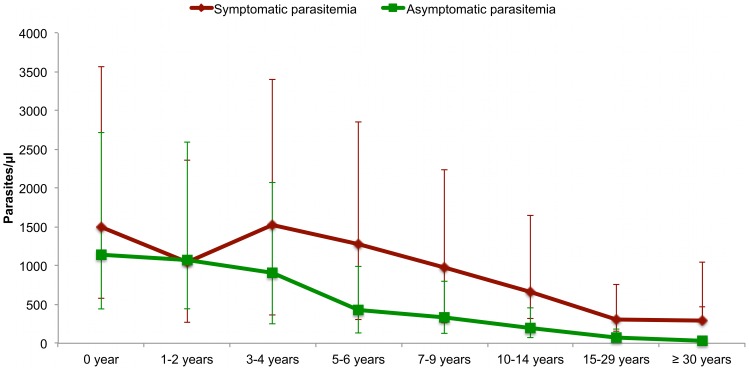
The mean *P. malariae* parasitemia (arithmetic mean of parasites per µl of blood) in asymptomatic control and fever case observations by age group, Dielmo 1990–2010.

Fig. 6 shows the mean *P. malariae* symptomatic and asymptomatic parasitemias according to age group between 1990 and 2010. Considering the changes in *P. malariae* prevalence during the 20 years of follow up, the relationship between *P. malariae* parasitemia and the occurrence of fever was investigated separately during three different periods: from June 1990 to December 1994, from January 1995 to December 2004 and from January 2005 to December 2010 (Table 3).

**Table 3:** Relationship between *Plasmodium malariae* parasitemia and risk of fever.

Level of *P. malariae* parasitemia^a^	Number of cases								
	Fever			Asymptomatic		Total			
	n	%		n	%	n	OR	95% CI	p^b^
**1990–1994**									
0	2,141	(72.9)		13,635	(81.2)	15,776	1		-
<30	338	(11.5)		1,606	(9.6)	1,944	1.6	[1.4–1.9]	**<0.001**
30–299	105	(3.6)		484	(2.9)	589	1.2	[1.0–1.6]	0.071
300–2999	275	(9.4)		955	(5.7)	1,230	1.4	[1.2–1.7]	**<0.001**
3000–5999	68	(2.3)		92	(0.5)	160	3.3	[2.3–4.7]	**<0.001**
≥6000	11	(0.4)		13	(0.1)	24	4.9	[1.9–13.1]	**0.001**
Total	2,938			16,785		19,723			
**1995–2004**									
0	8,293	(89.7)		18,427	(90.8)	26,720	1		-
<30	412	(4.5)		1,154	(5.7)	1,566	1.0	[0.9–1.2]	0.575
30–299	165	(1.8)		351	(1.7)	516	1.1	[0.9–1.3]	0.393
300–2999	253	(2.7)		328	(1.6)	581	1.5	[1.2–1.7]	**<0.001**
3000–5999	94	(1.0)		23	(0.1)	117	6.3	[3.9–10.2]	**<0.001**
≥6000	27	(0.3)		6	(0.03)	32	6.7	[2.7–16.9]	**<0.001**
Total	9,244			20,289		29,533			
**2005–2010**									
0	2,574		(96.8)	12,102	(97.8)	14,676	1		-
<30	38		(1.4)	180	(1.5)	218	1.3	[0.9–2.0]	0.116
30–299	9		(0.3)	49	(0.4)	58	1.1	[0.5–2.3]	0.848
300–2999	27		(1.0)	33	(0.3)	60	4.1	[2.4–7.2]	**<0.001**
3000–5999	8		(0.3)	4	(0.03)	12	8.7	[2.4–31.2]	**0.001**
≥6000	3		(0.1)	1	0.01)	4	15.6	[1.5–164.1]	**0.022**
Total	2,659			12,369		15,028			

a Parasites/µl of blood.

b Fisher's exact test. p values shown in bold are significantly associated with an increased risk of fever.

Dielmo, 1990–1994, 1995–2004, 2005–2010.

During the first period (1990–1994), 797 (27.1%) of 2,938 fever cases occurred in patients infected by *P. malariae*. Of 16,785 observations in asymptomatic persons, 3,150 (18.8%) involved persons infected with *P. malariae*. They were more fever cases with *P. malariae* associated with *P. falciparum* and/or *P. ovale* (765 cases, 96.0%) than fever cases with *P. malariae* only (32 cases, 4.0%). The risk of fever increased moderately when *P. malariae* parasitemia was higher than 3,000/µl or 6,000/µl of blood. All infections were symptomatic when parasitemia was higher than 8,500/µl of blood. Maximum *P. malariae* parasitemia observed during a fever episode was 16,560/µl in a 4 year old child (second highest value: 13,600/µl in a 3 year old child) and the maximum parasitemia without documented symptoms was 8,400/µl in a 3 year old child.

During the second period (1995–2004), 951 (10.3%) of 9,244 episodes of fever occurred in patients infected by *P. malariae*. Of 20,289 observations in asymptomatic persons, 1,862 (9.2%) involved persons infected with *P. malariae*. They were more fever cases with *P. malariae* associated with *P. falciparum* and/or *P. ovale* (661 cases, 69.5%) than fever cases with *P. malariae* only (290 cases, 30.5%). The risk of fever increased 6-fold when *P. malariae* parasitemia was above 3,000/µl of blood (p<0.001). Above 6,000/µl of blood, only 6 infections (18.7%) were asymptomatic. Maximum *P. malariae* parasitemia observed during a fever episode was 31,840/µl in a 3 year old child (second highest value: 21,760/µl in a 12 year child) and the maximum parasitemia without documented symptoms was 8,960/µl in a 3 year old child.

During the third period (2005–2010), 85 (3.2%) of 2,659 episodes of fever occurred in patients infected by *P. malariae*. Of 12,369 observations in asymptomatic persons, 267 (2.2%) involved persons infected with *P. malariae*. There was little difference between the number of fever cases associated with *P. malariae* in mixed infections (41, 48.2%) and the number of fever cases with *P. malariae* only (44, 51.8%). The risk of fever increased 9-fold when *P. malariae* parasitemia was above 3,000/µl of blood. Maximum *P. malariae* parasitemia observed during a fever episode was 8,240/µl in a 7 year old child (second highest value: 6,240/µl in a 11 year child) and the maximum parasitemia without documented symptoms was 6,560/µl in a 9 year old child.

**Figure 7:**
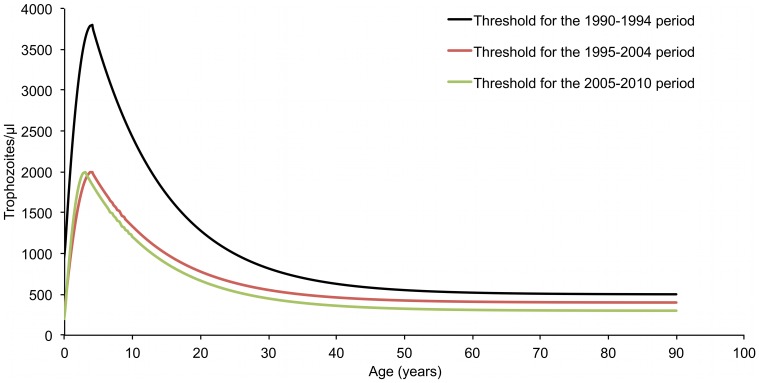
Random-effect logistic regression model derived threshold level of parasitemia for attributing fever episodes to *P. malariae* by age and period, Dielmo, 1990–2010.

The age-dependent pyrogenic threshold levels of *P. malariae* parasitemia during the three periods 1990–1994, 1995–2004 and 2005–2010 are shown in Fig. 7. Estimates of the parameters defining these thresholds are presented in Table 4. During the first period, highest threshold parasitemia in 4 year old children was 3,800/µl and lowest threshold parasitemia in adults was 500/µl. During the second period, highest threshold parasitemia in 4 year old children was 2,000/µl and lowest threshold parasitemia in adults was 400/µl. During the third period, highest threshold parasitemia in 3 year old children was 2,000/µl and lowest threshold parasitemia in adults was 300/µl.

**Table 4:** Estimates of the parameters defining the age-dependent pyrogenic threshold of *P. malariae* infections during the periods 1990–2004, 1995–2004 and 2005–2010.

Parameters	Period 1990–1994	Period 1995–2004	Period 2005–2010
Number of individuals	401	538	558
Number of case observations	2,938	9,244	2,659
Number of control observations	16,785	20,289	12,369
Exponent for the function of parasitemia (*r*)	0.04	1	0.43
Age in years of maximum parasitemia (*a*)	4	4	3
Maximum threshold parasitemia (*b*)	3800	2000	2000
Parasitemia at year 0 (*c*)	950	300	200
Lowest threshold parasitemia in adults (*d*)	500	400	300
Decay parameter of the function (*e*)	0.1	0.09	0.1
Threshold effect odds ratios (95% CI)	2.4 (1.7–3.4)	2.9 (1.8–4.5)	2.9 (0.8–10.3)
Continuous effect of parasitemia odds ratios for an increase of 100 parasites/µl (95% CI)	1.4 (1.2–1.5)	1.0 (0.9–1.1)	1.0 (0.9–1.1)
0–1 year old effect odds ratios	1.00	1.00	1.00
2–6 years old effect odds ratios (95% CI)	1.5 (1.3–1.8)	2.1 (1.9–2.3)	1.1 (0.9–1.2)
7–12 years old effect odds ratios (95% CI)	1.2 (0.9–1.5)	1.7 (1.5–1.9)	0.6 (0.5–0.7)
≥13 years old effect odds ratios (95% CI)	0.4 (0.3–0.5)	0.9 (0.8–1.1)	0.3 (0.2–0.3)
AIC	14306.8	32919.0	13149.0

### *P. ovale* and *P. malariae* clinical attacks

Over the 20 years of study, there were 22,266 episodes of fever or fever-related symptoms during the 2,110,321 person-days of clinical monitoring of the study population (children: 1,013,054 days; adults: 1,097,267 days). 219 clinical malaria attacks were attributable to *P. ovale* and 290 to *P. malariae*.

**Figure 8:**
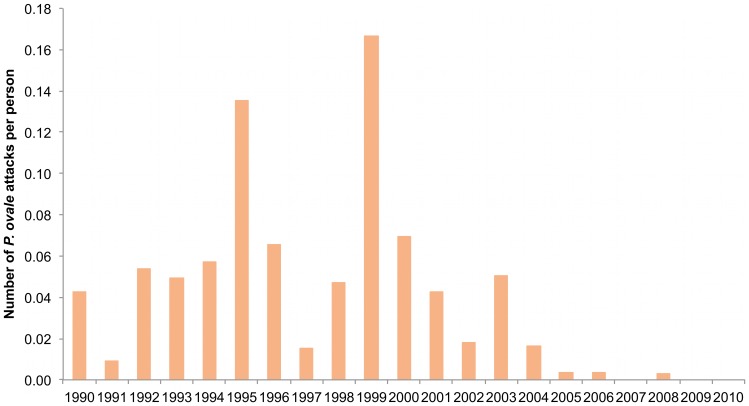
Trends in yearly incidence density of *P. ovale* clinical attacks. Dielmo, 1990–2010.

**Figure 9:**
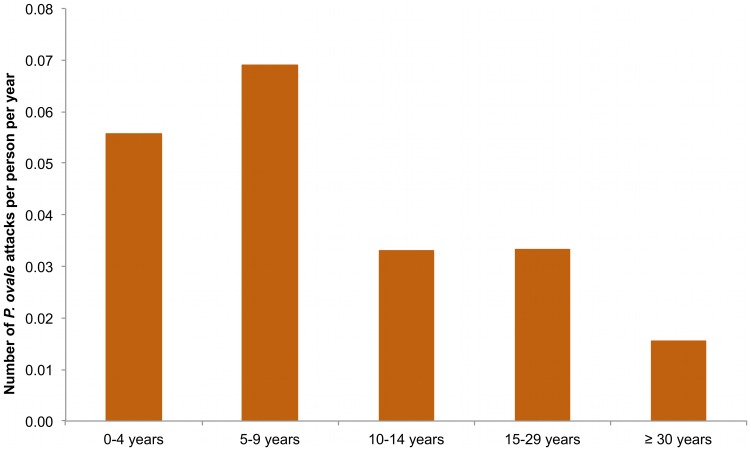
Mean yearly incidence density of *P. ovale* clinical attacks by age group. Dielmo, 1990–2010.

*P. ovale* attacks were observed in 155 persons from all compounds of the village (1, 2, 3, 4 and 5 attacks in 106, 38, 8, 2 and 1 villagers, respectively). Of the 38 individuals with two attacks, 18 presented their second attack within twelve months after the first attack (13 within six months). Of the 11 individuals with three or more attacks, 10 presented all attacks (5 individuals) or all attacks except one (5 individuals) within a twelve month period between two successive attacks, and one individual with three attacks had all attacks separated by at least two years each. The youngest child who presented *P. ovale* clinical malaria was a 2 month-old infant and the oldest was a 89 year old man. The median parasitemia reached during the 219 attacks was 8,426/µl. A temperature below 38°C (rectal) or 37.5°C (axillary) with fever-related symptoms only was observed in 40 attacks. A temperature ≥39°C was documented in 126 attacks. The average incidence density of *P. ovale* attacks at the community level from 1990 to 2010 was 0.04 attacks per person per year. Fig. 8 shows that the annual incidence density of *P. ovale* attacks was maximum in 1999, reaching 0.17 attacks per person per year, and minimum in 2007, 2009 and 2010 where no attack was observed. Fig. 9 shows the incidence density of *P. ovale* clinical attacks according to age during the whole study period. 151 of 219 cases (68.9%) occurred in children under 15 years-old and the mean incidence density was maximum in children 5–9 years where it averaged 0.07 attacks per child per year. Maximum incidence density in a given year and age group was observed in 1999 in children 0–4 year who presented 0.32 attacks per child. A total of 68 of 219 cases (31.1%) occurred in adults, including 28 cases (12.8%) in adults ≥30 years old. The incidence density was only 2.4 fold lower in adults ≥15 year (0.023 per year) than in children <15 year (0.054 per year). *P. ovale* attacks occurred all year round, without marked seasonal pattern (maximum: 30, 28 and 25 cases in October, February and November, respectively; minimum: 13 cases in March, August and September).

**Figure 10:**
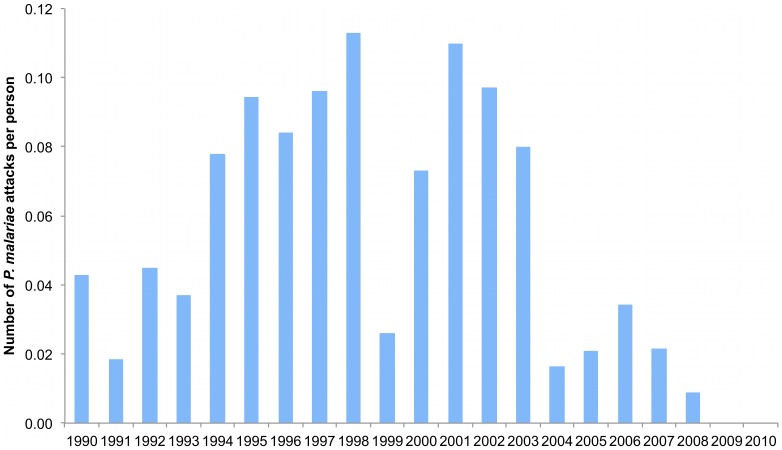
Trends in yearly incidence density of *P. malariae* clinical attacks. Dielmo, 1990–2010.

**Figure 11:**
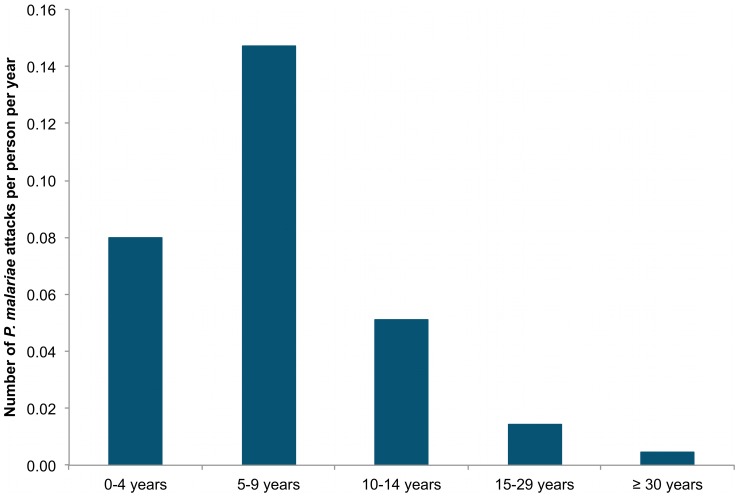
Mean yearly incidence density of *P. malariae* clinical attacks by age group. Dielmo, 1990–2010.

*P. malariae* attacks were observed in 131 persons from all compounds of the village (1, 2, 3, 4, 5, 6 attacks in 62, 29, 20, 8, 4, 6 villagers, respectively, and 9 and 13 attacks in one villager). Of the 29 individuals with two attacks, 16 presented their second attack within twelve months after the first attack (13 within six months). Of the 40 individuals with three or more attacks, 34 presented all attacks (16 individuals) or all attacks except one (18 individuals) within a twelve month period between two successive attacks, and six individuals with three attacks or more had from three to five attacks separated by at least one year. The youngest child who presented *P. malariae* clinical malaria was a 2 month-old infant and the oldest was a 42 year old man. The median parasitemia reached during the 290 attacks was 4,167/µl. A temperature below 38°C (rectal) or 37.5°C (axillary) with fever-related symptoms only was observed in 34 attacks. A temperature ≥39°C was documented in 188 attacks. The average incidence density of *P. malariae* attacks at the community level from 1990 to 2010 was 0.05 attacks per person per year. Fig. 10 shows that the annual incidence density of *P. malariae* attacks was maximum in 1998, reaching 0.11 attacks per person per year, and minimum in 2009 and 2010 where no attack was observed. Fig. 11 shows the incidence density of *P. malariae* clinical attacks according to age during the whole study period. 265 of 290 cases (91.4%) occurred in children under 15 years-old and the mean incidence density was maximum in children 5–9 years where it averaged 0.15 attacks per child per year. Maximum incidence density in a given year and age group was observed in 2002 in children 5–9 year who presented 0.39 attacks per child. A total of 25 of 290 attacks (8.6%) occurred in adults, including 8 attacks (2.8%) in adults ≥30 years old. The incidence density was 12 fold lower in adults ≥15 year (0.008 per year) than in children <15 year (0.096 per year). *P. malariae* attacks occurred all year round, without marked seasonal pattern (maximum: 32, 30 and 29 cases in March, August and May, respectively; minimum: 15, 20 and 20 cases in June, July and September, respectively).

## Discussion

Few studies have attempted to measure the burden *P. ovale* and *P. malariae* in malaria endemic areas of tropical Africa. Although many comprehensive studies have investigated *P. falciparum* malaria both in patients attending clinics and in cohort population studies where the occurrence of clinical malaria episodes was closely monitored, generally no information was published regarding data collected on *P. ovale* and *P. malariae* during these studies. Several reasons may explain the rarity of published works on these two malaria parasites, including the low incidence of the disease, and -due to the lack or rarity of severe cases- its much lower importance than *P. falciparum* in terms of public health, but also the practical difficulties of such studies that need specifically trained microscopists (young ring forms of the three species may be difficult to distinguish in thick blood films, especially during co-infections) and may require specific data analysis methods since *P. ovale* and *P. malariae* are often associated with *P. falciparum* at various but often very low levels of parasitemia.

The longitudinal study conducted in Dielmo during 20 years shows dramatic changes in the prevalence of the three malaria species between 1990 and 2010. Prevalence was high the first years of the project and declined to very low values in the most recent years. However, trends differed significantly according to species, especially for *P. ovale* between 1990 and 2004, which clearly presented strong annual variations independent of treatment policies. It would have been interesting to know if both (sub) species of the *P. ovale* complex (*wallikeri* and *curtisi* [24]) were involved in these annual variations but our preliminary retrospective PCR investigations with preserved stained thick blood films were unsuccessful to clarify this point. After 2004, *P. ovale* almost disappeared and *P. malariae* became much rarer, probably in relation to the switch from chloroquine to amodiaquine + SP as first line treatment of malaria attacks, although *P. ovale* and *P. malariae* infections have always been sensitive to chloroquine and other antimalarials. Unfortunately, *P. ovale* and *P. malariae* specific entomological inoculation rates were only monitored during the first years of the study [8], due to the lack of availability of good quality monoclonal antibodies for these species the following years. Between 1990 and 1992, 15.5% of *A. gambiae s.l*. and *A. funestus* mosquitoes with sporozoites in their salivary glands detected by dissection were infected by *P. malariae* alone or in association with *P. falciparum* and/or *P. ovale* and 8.2% were infected by *P. ovale* [8].

In malaria endemic areas, where asymptomatic infections are highly prevalent, the detection of malaria parasites in persons with fever is not sufficient criteria for distinguishing malaria from other causes of fever. Methods based on parasite density are widely used to confirm or discard the diagnosis of *P. falciparum* clinical malaria and to assess the burden of malaria [21], [22], [25]–[27], but these methods have never been applied to *P. malariae* and to *P. ovale*. To our knowledge the only comprehensive study of *P. malariae* morbidity in tropical Africa is the work of Miller [16] in Liberia who investigated the level of parasitemia associated with fever in a cohort of 20 adults aged from 20 to 30 years and 10 children aged from 3 to 7 years. Seven attacks were attributed to *P. malariae* during the study period, including two among adults with parasitemia ranging from 22 to 136/µl (mean: 79/µl) and five among children with parasitemia ranging from 1,650 to 5,935/µl (mean: 3,172/µl). In our study, in contrast to *P. falciparum* and *P. ovale*, *P. malariae* attacks in each age group were often associated with levels of parasitemia only scarcely higher than levels of parasitemia commonly observed during asymptomatic infections. In the case of *P. ovale*, we presented in a previous paper an analysis of the relationship between parasitemia and fever in Dielmo during the period 1990 to 1996 using a case control approach [19]. Results indicated that only parasite densities ≥800/µl were significantly associated with clinical symptoms. A constant threshold value of 800/µl for all age-groups during 20 years would have given 261 clinical *P. ovale* attacks, i.e. 42 attacks more than the 219 attacks measured by the age-dependent threshold-effect model, most of these additional attacks occurring in children under 10 years.

The incidence of *P. ovale* and *P. malariae* attacks in Dielmo population was much lower than the incidence of *P. falciparum* attacks. Using a similar model, 7,978 *P. falciparum* malaria attacks were diagnosed between October 1990 and December 2010 [22]. All *P. ovale* and *P. malariae* attacks were mild and of short duration, but fever was often high and one *P. ovale* attack was responsible for a stillbirth. *P. malariae* was more involved than *P. ovale* in mixed infections with *P. falciparum*, even at high parasitemia. There were 7 attacks due to *P. ovale* and 3 attacks due to *P. malariae* within 15 days of a *P. falciparum* clinical attack.

Although an analysis of interaction between malaria species and the presumed respective roles of species-specific and cross immunity is out of the scope of this paper, some observations are of interest. Both *P. ovale* and *P. malariae* first infections and attacks in infants were observed during the third month of life, although the mothers of these infants have spent much of their life under high transmission conditions, suggesting that materno-transmitted immunity was insufficient to prevent infection and disease, this in particular when there was no pre-existing *P. falciparum* infection. In children, the incidence of *P. ovale* and *P. malariae* attacks was much lower than expected from data on incidence *(P. ovale*) or prevalence (*P. malariae*) of patent infections as measured by bi-weekly, weekly or monthly microscopy [8], and this was particularly the case during the first years of the project when the prevalence of *P. falciparum* asymptomatic infections was maximum. The duration of patent infections was much shorter for *P. ovale* than for *P. malariae* and the dynamics of parasitemia differed (even when considering than microscopic examination underestimates parasite prevalence compared to PCR studies [28], [29]), clearly indicating that only a low proportion of new infections in a low number of individuals each year were responsible for a clinical attack, and suggesting a protective role of co-infections with *P. falciparum*. In fact most *P. ovale* clinical attacks occurred in individuals free of malaria parasites the previous days or weeks (independently of *P. falciparum* malaria treatments), but this was less the case for *P. malariae* attacks. Furthermore, no marked peak of parasitemia was observed for most *P. malariae* attributed clinical attacks, and since most *P. malariae* infections were long lasting with often high levels of chronic parasitemia in several children, it remains unclear to what extent our model was able to provide accurate measurements of *P. malariae* morbidity for all individuals. The two children who were attributed 13 and 9 *P. malariae* attacks were permanent resident of Dielmo and suffered these attacks between ages 2–13 years and 7–13 years, respectively.

Almost one third (31.1%) of *P. ovale* clinical attacks occurred among adults, versus only 8.6% for *P. malariae*. For both species a high proportion of clinical attacks occurred among permanent residents of Dielmo. On average, the mean incidence density of clinical attacks in adults was 2.9 fold higher for *P. ovale* (0.023 attacks per person per year) than for *P. malariae* (0.008 attacks per person per year). These results suggest that at least for some individuals continuously exposed since birth to many reinfections by *P. ovale* and *P. malariae*, acquired immunity may be lost or insufficient to prevent any clinical attack. However, as also observed for *P. falciparum* attacks, the duration of fever and other symptoms rarely exceeded one or two days, even when no antimalarials were given [30].

Chronic nephrotic syndromes attributed to *P. malariae* have been reported in the literature [5], [31], [32]. They carry a high rate of mortality both in children and adults, but this association remains at least in part controversial and was not specifically investigated in our study. During the 20-year period of surveillance, 60 deaths occurred in Dielmo villagers, including two deaths with a renal failure which were unlikely related to *P. malariae* (one men and one women aged 78 and 71 years, respectively). In the case of *P. ovale*, life-threatening illness may also occur at least occasionally, as recently reported in a non-immune traveler returning from Nigeria [33].

Although representing together only 5.9% of the whole malaria morbidity among Dielmo villagers, *P. ovale* and *P. malariae* were a relatively common cause of morbidity in most age groups, including adults, until the recent dramatic decrease of malaria that followed the introduction of new control policies combining ACTs and LLINs. *P. ovale* and *P. malariae* may remain an important cause of morbidity in many areas of tropical Africa.
